# *In vitro* Study of Bedaquiline Resistance in *Mycobacterium tuberculosis* Multi-Drug Resistant Clinical Isolates

**DOI:** 10.3389/fmicb.2020.559469

**Published:** 2020-09-17

**Authors:** Giulia Degiacomi, José Camilla Sammartino, Virginia Sinigiani, Paola Marra, Alice Urbani, Maria Rosalia Pasca

**Affiliations:** ^1^Laboratory of Molecular Microbiology, Department of Biology and Biotechnology Lazzaro Spallanzani, University of Pavia, Pavia, Italy; ^2^Istituto Universitario di Studi Superiori-IUSS, Pavia, Italy

**Keywords:** *Mycobacterium tuberculosis*, bedaquiline, multi-drug resistance, Rv0678, MmpL5, AtpE

## Abstract

Tuberculosis (TB) is one of the major causes of death related to antimicrobial resistance worldwide because of the spread of *Mycobacterium tuberculosis* multi- and extensively drug resistant (multi-drug resistant (MDR) and extensively drug-resistant (XDR), respectively) clinical isolates. To fight MDR and XDR tuberculosis, three new antitubercular drugs, bedaquiline (BDQ), delamanid, and pretomanid were approved for use in clinical setting. Unfortunately, BDQ quickly acquired two main mechanisms of resistance, consisting in mutations in either *atpE* gene, encoding the target, or in *Rv0678*, coding for the repressor of the MmpS5-MmpL5 efflux pump. To better understand the spreading of BDQ resistance in MDR- and XDR-TB, *in vitro* studies could be a valuable tool. To this aim, in this work an *in vitro* generation of *M. tuberculosis* mutants resistant to BDQ was performed starting from two MDR clinical isolates as parental cultures. The two *M. tuberculosis* MDR clinical isolates were firstly characterized by whole genome sequencing, finding the main mutations responsible for their MDR phenotype. Furthermore, several *M. tuberculosis* BDQ resistant mutants were isolated by both MDR strains, harboring mutations in both *atpE* and *Rv0678* genes. These BDQ resistant mutants were further characterized by studying their growth rate that could be related to their spreading in clinical settings. Finally, we also constructed a data sheet including the mutations associated with BDQ resistance that could be useful for the early detection of BDQ-resistance in MDR/XDR patients with the purpose of a better management of antibiotic resistance in clinical settings.

## Introduction

According to the World Health Organization (WHO) report, in 2018, tuberculosis (TB), caused by *Mycobacterium tuberculosis*, was one of the major causes of death related to antimicrobial resistance (World Health Organization [WHO], 2019a). Globally, in 2018 about half a million TB infections were rifampicin-resistant, of which 78% were multi-drug resistant (MDR)-TB (World Health Organization [WHO], 2019a). Among these cases, 6.2% were estimated to have extensively drug-resistant (XDR)-TB (World Health Organization [WHO], 2019a). Even if it is a relatively small percentage of all MDR-TB cases, these infections are more complicated to treat and to manage and are a challenge for the health systems worldwide.

Recently, three new antitubercular drugs, bedaquiline (BDQ) (Janssen, Beerse, Belgium), delamanid (Otsuka, Tokyo, Japan), and pretomanid (TB Alliance) were approved for the treatment of MDR-TB (Li et al., 2019; Nieto Ramirez et al., 2020). Interestingly, several studies demonstrated that patients treated with a BDQ-containing regimen showed a high culture conversion rate (65–100%) (Li et al., 2019; Pontali et al., 2019).

BDQ is a diarylquinoline that targets *atpE* gene, coding for the subunit c of the ATP synthase complex (Andries et al., 2005). Its use reduces the mortality when added to treatment for MDR- and XDR-TB (Li et al., 2019; Conradie et al., 2020). The potential risk of BDQ of prolonging the QT interval has occurred in only 0.6% of treated patients; consequently, the advantage in its use is uncontested, even if it is still under investigation. In fact, many clinical studies are testing the effectiveness of new drug combinations, which include BDQ, to design the next generation regimens (Sharma et al., 2020).

In this context, WHO has recently updated the treatment for MDR-TB, by recommending two possible regimens (the longer regimen and the shorter one), both including BDQ and other drugs (Caminero et al., 2019; World Health Organization [WHO], 2019b). Interestingly, in a recent study, NIX-TB trial, a three-drug regimen including linezolid, BDQ and pretomanid was tested with XDR- and MDR-TB patients; the therapy was successful for 90% of patients (Conradie et al., 2020). As evident, BDQ use is rapidly spreading, and 90 countries reported having imported or started using BDQ by the end of 2018 (World Health Organization [WHO], 2019a).

In spite of its recent use in clinical practice, primary BDQ resistance appeared among *M. tuberculosis* clinical isolates (Veziris et al., 2017; Villellas et al., 2017; Zimenkov et al., 2017; Ismail et al., 2018). BDQ resistance is especially associated with mutations in *atpE* and *Rv0678* genes.

The most common mutations linked to low-level of BDQ resistance are present in *Rv0678* gene coding for the *M. tuberculosis* repressor of MmpS5-MmpL5 efflux system. This transporter pumps out of the cells also clofazimine and azoles (Milano et al., 2009; Hartkoorn et al., 2014; Smith et al., 2017). In some cases, *Rv0678* mutations occurred together with polymorphisms in other genes encoding the uncharacterized transporter Rv1979c and the cytoplasmic peptidase PepQ (Rv2535c), both associated with cross-resistance to clofazimine (CFZ) (Nieto Ramirez et al., 2020). Furthermore, a report demonstrated that mutations in *pepQ* gene confer low-level of BDQ resistance in mice (Almeida et al., 2016).

As expected, high BDQ resistance levels are caused by mutations in *atpE* gene, even if their frequency is extremely low among TB patients (Nieto Ramirez et al., 2020).

The surveillance of drug resistance during clinical management is mandatory in order to prevent the occurrence of BDQ resistance among TB patients. To this aim, *in vitro* studies could be a valuable tool for understanding the reasons linked to the spreading of BDQ resistance in particular amongst *M. tuberculosis* MDR and XDR clinical isolates. While acquiring resistance to first-line drugs such as rifampicin (RIF) and isoniazid (INH) is linked to a perturbance in the *M. tuberculosis* fitness (Kodio et al., 2019), mutations in *Rv0678* and *atpE* have not been yet demonstrated to have this behavior (Andries et al., 2014; Nieto Ramirez et al., 2020). On the other hand, the low frequency of *atpE* mutants in the clinical setting in comparison to *Rv0678* mutations could suggest a possible reduced fitness cost linked to some *atpE* mutations (Nieto Ramirez et al., 2020).

To better understand the spreading of BDQ resistance in MDR- and XDR-TB, we reported an *in vitro* generation of *M. tuberculosis* mutants resistant to BDQ starting from MDR clinical isolates as parental cultures, since BDQ is used to treat patients affected by MDR-TB. Moreover, we performed growth curves of both obtained BDQ resistant mutants and original MDR isolates to detect possible differences in strains harboring either *Rv0678* or *atpE* mutations. Furthermore, we compared these mutations to a compiled data sheet of previously published SNPs, deriving from *in vitro*, *in vivo* and clinically resistant strains, thus providing additional information for rapid and efficient detection of all known BDQ-resistance associated mutations to ensure an optimal treatment monitoring.

## Materials and Methods

### Bacterial Strains, Growth Conditions and Drugs

*Mycobacterium tuberculosis* H37Rv and clinical isolates as well as their mutants were grown at 37°C in Middlebrook 7H9 broth (Becton Dickinson), supplemented with 0.05% w/v Tween 80 or on Middlebrook 7H11, both supplemented with 0.2% w/v glycerol, and 10% v/v Middlebrook OADC enrichment (oleic acid, albumin, D-glucose, catalase; Becton Dickinson). *M. tuberculosis* MDR clinical isolates were collected and characterized at the Sondalo Division of the Valtellina and Valchiavenna, Italy, hospital authority in 2012 (Menendez et al., 2013). Bedaquiline (D.B.A. Italia s.r.l.) was dissolved in DMSO (Sigma Aldrich).

All the experiments with *M. tuberculosis* were performed in Biosafety level 3 laboratory by authorized and trained researchers.

### Genomic DNA Preparation and Whole-Genome Sequencing of *M. tuberculosis* Clinical Isolates

Genomic DNA of *M. tuberculosis* MDR clinical isolates (hereafter named IC1 and IC2) was extracted as previously described (Belisle and Sonnenberg, 1998). Genomic DNA samples were sequenced by using an Illumina HiSeq2000 technology at Fisabio (Valencia, Spain). Illumina reads were aligned to the annotated genome sequence of the wild-type H37Rv (Cole et al., 1998) (NC_000962.3) to identify SNPs. For the bioinformatic analysis of Illumina data, repetitive PE and PPE gene families were discarded as well as SNPs and indels with less than 50% probability. The possible polymorphisms associated to the resistance to the following drugs were investigated: streptomycin (*rrs*, *rpsL*, *gidB*), INH (*katG*, *inhA*, *ndh*, *nat*), RIF (*rpoB*), ethambutol (*embA*, *embB*, *embC*, *embR*), ethionamide (*ethA*, *inhA*, *ethR*), pyrazinamide (*pncA*, *rpsA*, *panD*), capreomycin (*tlyA*, *rrs*), and BDQ (*Rv0678*, *atpE*, *pepQ*).

### Determination of Minimal Inhibitory Concentration (MIC)

The drug susceptibility of *M. tuberculosis* strains was determined using the resazurin microtiter assay (REMA), as previously described (Palomino et al., 2002). Briefly, log-phase bacterial cultures were diluted to a theoretical OD_600_ = 0.0005 and grown in a 96-well black plate (Fluoronunc, Thermo Fisher) in the presence of serial compound dilution. A growth control containing no compound and a sterile control without inoculum were also included. After 7 days of incubation at 37°C, 10 μl of resazurin (0.025% w/v) were added and fluorescence was measured after 24 h further incubation using a Fluoroskan^TM^ Microplate Fluorometer (Thermo Fisher Scientific; excitation = 544 nm, emission = 590 nm). Bacterial viability was calculated as a percentage of resazurin turnover in the absence of compound.

### Isolation and Characterization of *M. tuberculosis* Spontaneous Mutants Resistant to BDQ

*Mycobacterium tuberculosis* BDQ resistant mutants were isolated by plating approximately 10^8^ and 10^9^ CFU from exponential growth phase cultures of IC1 and IC2 clinical isolates onto solid medium containing drug at concentrations exceeding the MIC (5X, 10X, 20X MIC). Following 6–8 weeks of incubation, BDQ resistant colonies were streaked onto 7H11 medium. At the same time, these colonies were streaked also onto 7H11 medium plus the same BDQ concentration used for mutant isolation to confirm the resistant phenotype. BDQ MIC values were also assessed by REMA. Genomic DNA was extracted from each mutant and *Rv0678*, *atpE*, and *pepQ* genes were amplified by PCR (oligonucleotides in Supplementary Table S1), purified using Wizard^®^ SV Gel and PCR Clean-Up System (Promega) and analyzed by conventional Sanger sequencing (Eurofins Genomics, Italy).

### Growth Curves of *M. tuberculosis* BDQ Resistant Mutants and MDR Clinical Isolates

The cultures of *M. tuberculosis* mutants, as well as their corresponding parental strain, were inoculated in 7H9 medium in round bottom tubes at 37°C to reach an early exponential phase. Then, each strain was reinoculated in new 7H9 medium at final OD_600_ = 0.06. The cultures were incubated in standing at 37°C for 8 days. After 24, 48, 96, 168, 192 h, the optical densities at 600 nm were recorded to plot growth curves. The H37Rv strain was also included as control.

### Data Sheet Creation

The Medical Subject Headings vocabulary of biomedical terms (MeSH) search builder was used to construct the query for the terms “Bedaquiline,” “*Mycobacterium*,” and “Mutation”, with which the Pubmed and Pubmed Central databases were skimmed, then all the abstracts were downloaded in a MEDLINE format. The information gathered was then manually filtered in two categories: relevant papers (i.e., original works, case studies, and clinical studies) and papers not pertinent to our purpose. The filtered-as-relevant papers were downloaded as full text, thoroughly analyzed and the type of mutations linked to BDQ resistance were annotated to set-up the data sheet.

## Results

### Characterization of *M. tuberculosis* MDR Clinical Isolates

IC1 and IC2 strains are two *M. tuberculosis* MDR clinical isolates previously characterized (Menendez et al., 2013). In detail, IC1 is resistant to streptomycin (SM), INH, RIF, ethambutol (EMB), ethionamide (ETH); IC2 is resistant not only to the previously mentioned drugs, but also to pyrazinamide (PYR), and capreomycin (CM) (Menendez et al., 2013).

REMA was used to determine the BDQ MIC values of both isolates (MIC = 0.06 μg/ml, as for the H37Rv wild-type strain). This MIC value (0.06 μg/ml) for *M. tuberculosis* BDQ sensitive strains is in agreement with that proposed in 7H9 medium by both EUCAST and previously (Kaniga et al., 2016; EUCAST, 2020).

In order to pinpoint the SNPs responsible for their drug-resistance profile, the *M. tuberculosis* clinical strains were subjected to whole-genome sequencing (WGS) analysis. Using the obtained Illumina data, the genes involved in the resistance to the following drugs were checked: SM (*rrs*, *rpsL*, *gidB*), INH (*katG*, *inhA*, *ndh*, *nat*), RIF (*rpoB*), EMB (*embA*, *embB*, *embC*, *embR*), ETH (*ethA*, *inhA*, *ethR*), PYR (*pncA*, *rpsA*, *panD*), CAP (*tlyA*, rrs), and BDQ (*Rv0678*, *atpE*, *pepQ*).

For both *M. tuberculosis* isolates, the non-synonymous mutations found to be associated to their drug-resistance phenotype are enlisted in Table 1. As expected, no mutation was found in *Rv0678*, *atpE*, and *pepQ* genes according to their BDQ sensitivity.

**TABLE 1:** Phenotypic and genotypic characteristics of *M. tuberculosis* IC1 and IC2 clinical isolates.

**Resistance**	**Genome position (bp)**	**Gene**	**Mutation**	**Amino acid substitution**
**Clinical isolate IC1**				
STR	4407880	*gidB*	T323G	L108R
INH	2155168	*katG*	G944C	S315T
RIF	761155	*rpoB*	C1349T	S450L
EMB	4242803	*embC*	G2941C	V981L
EMB	4247429	*embB*	A916G	M306V
ETH	4327058	*ethA*	G416A	G139D
**Clinical isolate IC2**				
STR	781687	*rpsL*	A128G	K43R
INH	2155168	*katG*	G944C	S315T
RIF	761155	*rpoB*	C1349T	S450L
EMB	4242803	*embC*	G2941C	V981L
EMB	4247429	*embB*	A916G	M306V
PYR	2288807	*pncA*	C435G	D145E
PYR	2289106	*pncA*	G136A	A46T
ETH	4327058	*ethA*	G416A	G139D
CM	1918707	*tlyA*	insAG	Frameshift

*Mycobacterium tuberculosis* IC1 and IC2 clinical strains were used for further experiments because of their drug-resistance phenotype as well as their BDQ sensitivity.

### Isolation and Phenotypic Characterization of Spontaneous *M. tuberculosis* Mutants Resistant to BDQ

Once shown their BDQ sensitivity, *M. tuberculosis* IC1 and IC2 clinical isolates were used to select and to isolate BDQ-resistant spontaneous mutants, since patients affected by MDR-TB are likely to receive BDQ as part of their therapy.

Mutants were selected onto solid medium containing high BDQ concentrations (0.3, 0.6, 1.2 μg/ml, corresponding to 5, 10, 20-fold MIC, respectively). *Mycobacterium tuberculosis* BDQ-resistant mutants were isolated at a frequency of about 1.8 × 10^–8^ for IC1 and 6 × 10^–9^ for IC2.

All the 12 isolated mutants showed to be BDQ resistant and their MIC value was confirmed by REMA, ranging from 0.25 μg/ml (4X MIC of sensitive strain) to 8 μg/ml (128X MIC of sensitive strain) (Table 2). The different levels of drug-resistance could be linked to different associated mutations.

**TABLE 2:** Phenotypic and genotypic characteristics of mutants resistant to BDQ obtained from *M. tuberculosis* clinical isolates IC1 and IC2.

***M. tuberculosis* strains**	**MIC (μg/ml)**	**Mutation**	**Amino acid substitution**
H37Rv	0.06		
IC1	0.06		
IC2	0.06		
IC1 B	8	*atpE*: g187c	A63P
IC1 C	4	*atpE*: g187c	A63P
IC1 D	4	*atpE*: g187c	A63P
IC1 F	4	*atpE*: g187c	A63P
IC1 G	4	*atpE*: g187c	A63P
IC1 H	2	*atpE*: a83c	D28A
IC2 Q	0.5	*atpE*: a83g	D28G
IC1 L	0.5	*rv0678*: c400t	R124Stop
IC1 M	0.5	*rv0678*: g120c	L40F
IC1 N	0.5	*rv0678*: a271c	T91P
IC1 O	0.5	*rv0678*: g61t	E21Stop
IC2 P	0.25	*rv0678*: g197a	G66E

In order to investigate this possibility, *Rv0678*, *atpE*, and *pepQ* genes were amplified by PCR from the genomic DNA of *M. tuberculosis* BDQ resistant mutants and sequenced by Sanger method.

None of 12 *M. tuberculosis* resistant mutants had mutation in *pepQ* gene, while polymorphisms were found either in *atpE* or *Rv0678* genes (Table 2 and Supplementary Data Sheet S1).

In particular, seven strains carried a mutation in AtpE, the cellular BDQ target. Among them, five mutants harbored the same replacement of alanine at position 63 by a proline (A63P). IC2Q mutant had a substitution of the aspartic acid at position 28 with a glycine (D28G), while IC1H mutant presented at the same position a substitution with an alanine (D28A), (Table 2 and Supplementary Data Sheet S1). Furthermore, these mutants were characterized by a high level of BDQ resistance (2–8 μg/ml) ranging from 32 to 128X MIC of the wild-type strain.

The other five isolated *M. tuberculosis* mutants harbored mutations in *Rv0678*, encoding the MmpR transcriptional repressor of the efflux pump MmpS5-MmpL5. These mutants were characterized by a low level of BDQ resistance (0.25–0.5 μg/ml) corresponding to 4–8X MIC the wild-type strain. Three mutants, IC1M, IC1N, and IC2P, presented an amino acid change: respectively, the leucine at position 40 was replaced by a phenylalanine, the tyrosine at position 91 by a proline, and, finally, the glycine at position 66 by a glutamate (Table 2 and Supplementary Data Sheet S1). The MmpR of the other two mutants, IC1L and IC1O, was truncated by a stop codon at position 400 and at position 61, respectively. Additional experiments to demonstrate the role of Rv0678 in BDQ resistance were not performed. None of these *Rv0678* polymorphisms was already published, at the best of our knowledge.

### Evaluation of the Possible Influence of *atpE* and *Rv0678* Mutations to the Growth Rate of *M. tuberculosis* BDQ Resistant Mutants

**FIGURE 1:**
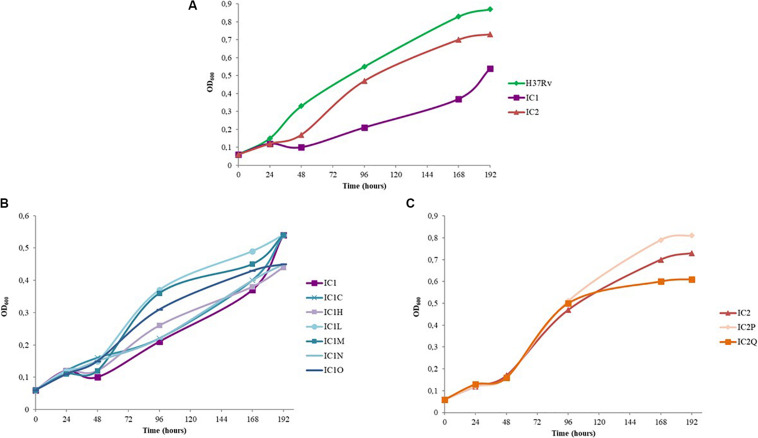
Growth curves of standing cultures of *M. tuberculosis* strains (H37Rv, clinical isolates and mutant strains). The experiment was repeated three times giving comparable results. The figure shows one representative experiment. **(A)** Growth curves of *M. tuberculosis* H37Rv strain and IC1 and IC2 clinical isolates. **(B)** Growth curves of IC1 clinical isolate and the following BDQ resistant mutants: IC1 C (representative mutant strain with A63P mutation in AtpE), IC1 H, IC1 L, IC1 M, IC1 N, IC1 O. **(C)** Growth curves of IC2 clinical isolate and the BDQ resistant mutants IC2 P and IC2 Q.

The growth curves of BDQ resistant mutants with respect to that of the *M. tuberculosis* H37Rv strain and the two parental MDR isolates were also evaluated (Figure 1).

As expected, IC1 and IC2 isolates presented a longer lag phase with respect to that of the wild-type strain. This is also in agreement with their lower growth rate (Figure 1A).

Interestingly, the rate of growth of IC2Q (*atpE* mutant) was lower than that of IC2 strain and of IC2P mutant (*Rv0678* mutant); on the other hand, IC2P grew faster than the other two strains (Figure 1C).

In the case of IC1-derived mutants, the lag phase length is similar between the mutants and the parental strain, while the rate of growth of IC1L, IC1M, IC1N, and IC1O (*Rv0678* mutants) was faster than that of IC1 (Figure 1B). These latter harbor mutations in *Rv0678* which do not perturb *M. tuberculosis* essential functions, whilst IC1C and IC1N (both *atpE* mutants) displayed a rate of growth similar to the parental one.

Overall, our data highlight that the *Rv0678* mutations do not affect growth rate of our parental strains, but could actually give an advantage in the growth rate.

### Collection of All Known Polymorphisms Causing BDQ Resistance in *M. tuberculosis* and Other Mycobacteria

The previously published mutations associated with BDQ-resistance as well as the new ones found in this work were included in Supplementary Data Sheet S1.

BDQ is also active against non-tuberculous mycobacteria (NTM) belonging to the *Mycobacterium avium-intracellulare* complex (MAC) and the *Mycobacterium abscessus* complex (MABSC) (Philley et al., 2015). Its possible use against these other mycobacterial species is under investigation. In NTM species BDQ presents the same mechanisms of resistance found in *M. tuberculosis*; consequently, the evaluation of the BDQ resistance associated polymorphisms could be useful also in these species. For this reason, in this collection, all the mycobacterial species in which an actual clinical use or possible use is under evaluation were included. Moreover, both *in vitro* isolated mutants and clinical isolates were added. At the end, all the mutations linked to BDQ resistance in mycobacteria have been considered in this data sheet that could be useful for the better understanding of BDQ resistance.

## Discussion

The current use of BDQ in the treatment for MDR- and XDR-TB reduces the mortality and it is highly effective (Ahmad et al., 2018; Conradie et al., 2020).

Nevertheless, the two main mechanisms of BDQ resistance, which are already widespread, are: modification of target (mutations in *atpE*, coding for the target) and over-expression of an efflux pump (mutations in *Rv0678* gene, coding for the repressor of MmpS5-MmpL5 efflux system). Several reports showed that the most spreading mechanism of BDQ resistance in clinical setting is represented by mutations in *Rv0678* gene even if with a low level of BDQ-resistance (Villellas et al., 2017; Nieto Ramirez et al., 2020). To fight BDQ-resistance caused by Rv0678 mutations and to allow its use for the largest possible part of patients, verapamil, an efflux inhibitor, could be the keystone, since it has been demonstrated to increase the efficacy of BDQ against both *M. tuberculosis* and *Mycobacterium abscessus* (Ghajavand et al., 2019; Viljoen et al., 2019).

In this study, an *in vitro* generation of *M. tuberculosis* mutants resistant to BDQ was performed starting from two MDR clinical isolates as parental cultures since patients affected by MDR-TB are eligible to receive BDQ as part of their therapy.

Polymorphisms were identified in both *Rv0678* and *atpE* genes. Our results confirm that these genes represent the main genetic drivers for the onset of BDQ-resistance, as previously pointed out.

Most *in vitro* isolated mutants harbored mutations in the BDQ target, AtpE at positions 28 and 63, according to previous multiple reports (Huitric et al., 2010; Segala et al., 2012; Zimenkov et al., 2017; Ismail et al., 2018). In particular, the mutation A63P was found in the first report regarding BDQ discovery (Andries et al., 2005). The 28 and 63 amino acid positions are considered mutation hotspots, as described in Supplementary Data Sheet S1. In fact, the AtpE D28 and A63 are not directly involved in the BDQ binding, but the disruption of the non-covalent bonds they form causes resistance (Preiss et al., 2015). Thanks to the previously published structure of complex crystals obtained by the co-crystallization of the *Mycobacterium phlei* c-ring with BDQ, it is well-known that BDQ forms an extensive amount of van derWaals interactions with a stretch of nine residues (in *M. phlei*: G62, L63, E65, A66, A67, Y68, F69, I70, and L72) provided by two adjacent *c*-subunits (Preiss et al., 2015). The mutations (D28A/G, A63P) harbored by BDQ-resistant mutants isolated in this study are positioned close to BDQ-binding site causing indirect structural interference with BDQ binding (Preiss et al., 2015), as evident by the higher MIC showed.

Different mutations could be linked to different levels of drug-resistance, as previously shown (Andries et al., 2005; Hartkoorn et al., 2014; Almeida et al., 2016). In general, *atpE* gene associated variants lead to high level of BDQ-resistance, but the most troublesome polymorphisms are linked to *Rv0678* gene, that are also the most represented ones found in clinical isolates even if the *Rv0678* mutations are linked to a lower level of BDQ resistance (Villellas et al., 2017; Nieto Ramirez et al., 2020), as typical for efflux pump mechanism. When over-expressed, MmpS5-MmpL5 efflux system can extrude different classes of drugs, for example CFZ and azoles, further limiting the therapeutic options of patients affected by M/XDR-TB. As well exemplified also in this present study, different mutations were identified in *Rv0678* and were disseminated across the gene (Table 2 and Supplementary Data Sheet S1). Both the missense mutations and the nonsense mutations are reported to abolish the repressor activity of Rv0678, causing an over-expression of the MmpL5 efflux pump leading to drug extrusion (Zhang et al., 2015). It is worth noting that a G66 missense mutation was found not only in this study (G66E), but also in CFZ-resistant *M. tuberculosis* mutant isolated *in vitro* (G66V) (Zhang et al., 2015).

As responsible for BDQ resistance, mutations in the intergenic region between *Rv0678* and *MmpS5* as well as mutations in the genes encoding the efflux pump MmpS5/MmpL5 were also reported (Ghajavand et al., 2019).

Furthermore, several reports showed that *Rv0678* mutations could be present prior the BDQ treatment both *in vitro* and *in vivo* (Pang et al., 2017; Veziris et al., 2017; Villellas et al., 2017; Xu et al., 2017; Chawla et al., 2018; Martinez et al., 2018). Consequently, it could be hypothesized that these mutations are adaptative or could improve the growth rate of the MDR mutants, representing an advantage for them. In this work, we evaluated the growth rate of our *M. tuberculosis* BDQ-resistant mutants in comparison with that of the parental strains (two MDR clinical isolates). The *atpE* mutants presented a growth rate similar or lower than that of the parental strains, since *atpE* is essential for *M. tuberculosis* growth, while *Rv0678* mutants showed either a similar growth rate as parental strains or better. Noteworthy, *Rv0678* gene is not essential for *M. tuberculosis* growth (Radhakrishnan et al., 2014). Finally, from our work the relative fitness of the mutants could be speculated. In fact, fitness cost determines in part the fate of resistance mutations (Melnyk et al., 2015). *In vitro* the *atpE* mutants seem to show a little decrease in fitness relative to that of the respective parental strain. On the opposite hand, *Rv0678* mutants seem to have the same fitness in comparison to the corresponding isolate or even a little advantage.

Previous studies showed that Rv0678 repressor controls the expression of MmpS5-MmpL5 efflux system as well as of other transporters such as IniAB and DrrA (Milano et al., 2009; Andries et al., 2014). Among the regulated proteins, there were also some essential enzymes and proteins important for the virulence (e.g., PimA, an antitoxin VapB1, etc.) (Andries et al., 2014), that could play a role in *M. tuberculosis* growth and/or infection. Apart from a genomic approach, proteomics-based approaches coupled with bioinformatics could be useful for the characterization of novel proteins which might be related to drug resistance, especially when no related mutations could explain it (Sharma et al., 2018).

## Conclusion

The presented *in vitro* growth rate data could explain the spreading of *Rv0678* naturally occurring mutations in clinical settings also prior BDQ treatment, even if we cannot exclude the presence of other compensatory mutations that alleviate the cost of resistance without altering it. This evidence also suggests a role for fitness in BDQ-resistance spread, even if further investigations are needed to clearly elucidate this mechanism.

Overall, due to an increasing BDQ usage, it is urgent to implement a more extensive surveillance for such resistance in order to prevent the emergence of resistance in clinical settings. Our collection of polymorphisms responsible for BDQ resistance could be used as theranostics targets, such as in the development of a diagnostic kit for the early detection of BDQ resistant isolates to better manage the available therapeutic options.

## Data Availability Statement

All datasets presented in this study are included in the article/Supplementary Material.

## Author Contributions

GD and MP designed this study and interpreted the data. GD, JS, and MP wrote the manuscript. GD, JS, VS, PM, and AU performed experiments. All authors approved the final version of the manuscript.

## Conflict of Interest

The authors declare that the research was conducted in the absence of any commercial or financial relationships that could be construed as a potential conflict of interest.
