# Supplementary material for: In vitro Study of Bedaquiline Resistance in Mycobacterium tuberculosis Multi-Drug Resistant Clinical Isolates
Source: Front Microbiol. 2020 Sep 17;11:559469. doi: 10.3389/fmicb.2020.559469 (PMC7527418; doi:10.3389/fmicb.2020.559469)
Supplement: Supplementary file 1 [file Table_1.PDF]

Supplementary Material

Supplementary Table

TABLE S1 | Primers used for DNA sequencing.

Primers	Sequence (5' to 3')	Purpose
GD19Rv0678SEQf	atgagcagcggtatccag	*rv0678* DNA sequencing
GD20Rv0678SEQr	atcgccgtcttgctcgcca	
GD21AtpESEQf	aacggctaccagagcca	*atpE* DNA sequencing
GD22AtpESEQr	ttcacccattgctaccta	
GD23pepQintSEQf	ctagcctgcgaagcagccg	*pepQ* DNA sequencing
GD24pepQintSEQr	atcagggcctccagctc	
GD25pepQf	cgcgcagcatccagttag	
GD26pepQr	ggtcgccacgtgggtct	
